# Sustainable milk-based postbiotics beverages fermented by *Lactobacillus plantarum*: allies in celiac disease inflammation

**DOI:** 10.3389/fnut.2025.1549120

**Published:** 2025-05-13

**Authors:** Claudia Bellomo, Francesca Mauriello, Federica Nigro, Francesca Passannanti, Rosa Colucci Cante, Roberto Nigro, Maria Vittoria Barone, Merlin Nanayakkara

**Affiliations:** ^1^Department of Translational Medical Science, Section of Pediatrics, University Federico II, Naples, Italy; ^2^ELFID (European Laboratory for the Investigation of Food Induced Diseases), University Federico II, Naples, Italy; ^3^Department of Chemical Engineering, Materials, and Industrial Production, University of Naples Federico II, Naples, Italy; ^4^I.T.P. Innovation and Technology Provider S.r.l., Naples, Italy; ^5^Department of Business Engineering Mario Lucertini, University of Tor Vergata, Rome, Italy; ^6^Federico II University Hospital, Naples, Italy

**Keywords:** celiac disease, postbiotic, CLA, Caco-2 cells, intestinal organoids, inflammation

## Abstract

**Background:**

Celiac disease (CeD) is an autoimmune disorder characterized by damage to the small intestine that occurs in genetically predisposed individuals after gluten consumption. Dietary exclusion is the only treatment. Gliadin is one of the main protein component of wheat gluten, and is poorly digested. Undigested peptide, p31-43, triggers several different processes, including inflammation. Intestinal organoids from CeD biopsies are good models for studying CeD inflammation. Postbiotics have been shown to modulate the effects of p31-43 in Caco-2 cells and inflammation in CeD organoids. The aims of this study was to study the anti-inflammatory activity of milk-based postbiotics from of *L. plantarum*.

**Methods:**

Postbiotics from *L. plantarum* CECT 749-fermented milk enriched with LA (linoleic acid), SCGs (Spent Coffee Grounds) and SCG oil were produced. Gliadin peptide p31-43 was used to induce inflammation on Caco2 cells. Organoids were derived from intestinal biopsies of 3 controls (CTRs) and 3 GCD (gluten containing diet)-CeD patients. NF-kB activation, a marker of inflammation, was evaluated by Western Blot analysis.

**Results:**

The results showed that pretreatment with all milk-based postbiotics of *L. plantarum*, except for SCG oil, inhibited the activation of NF-kB in the presence of the gliadin peptide in Caco-2 cells. The most efficient postbiotics, namely, milk-based postbiotics of *L. plantarum* with or without SCGs, could also reduce inflammation in intestinal organoids from CeD patients.

**Conclusion:**

Milk-based postbiotics of *L. plantarum*, with or without SCGs, prevents the proinflammatory effects of gliadin on Caco-2 cells and constitutive inflammation in CeD intestinal organoids, independent of the CLA (Conjugated linoleic acid) concentration.

## 1 Introduction

Celiac disease (CeD) is an immune-mediated enteropathy that primarily affects the small intestine in response to the ingestion of gluten, a protein present in wheat, barley, and rye. A gluten-free diet is the only therapy for these patients, who must eliminate gluten-containing cereals from their diet for life. CeD results from interactions between genetic factors, which are characterized mainly by HLAs, Human Leukocyte Antigen, class I and II genes, and environmental factors, which contribute to the generation of inflammation in the intestine (1–3). Among the environmental factors that induce inflammation, nutrients, including gluten, play a central role. Nutrients may have two effects, proinflammatory or anti-inflammatory effects, and the microbiota may mediate their destiny (4). However, how, when, and where inflammation is generated in CeD is still being determined (5).

Gliadin, the main protein in wheat-gluten, is a protein that is difficult to digest by intestinal endopeptidases. Undigested gliadin peptides may have biological activity (6). Some of them can activate the adaptive immune response, as they are well presented to T cells. Other peptides, such as the p31–43 peptide, play key roles in the inflammatory/innate immune response to gliadin (6). Specifically, this peptide has pleiotropic activity, including the induction of proliferation and inflammation with the activation of the nuclear transcription factor-B (NF-kB) pathway (7–9). The nuclear factor kappa B (NF-κB) family of transcription factors is a key regulator of immune development, immune responses, inflammation, and cancer. However, it is activated after phosphorylation mechanisms (pNF-kB). The activation of this pathway is associated with several stimuli, and upon ligand-receptor engagement, distinct cellular outcomes, appropriate to the specific signal received, are set into motion (10).

NF-kB is also a key protein in the regulation of inflammation in the intestine (11). Moreover, the NF-kB pathway has been found to be altered in intestinal biopsy samples from CeD patients, both at GCD (Gluten Containing Diet) and GFD (Gluten Free Diet) (12, 13), and in intestinal organoids (13). Interestingly, after gluten treatment, there was an increase in NF-kB expression in CeD biopsy samples at both GCD and GFD (1, 12).

Interestingly, in CeD biopsy samples and organoids, constitutive inflammation, characterized by an increase in IL6 and IL1B, which are also part of the NF-kB pathway, has been described even before the occurrence of the intestinal lesion (13, 14). The constitutive inflammation of intestinal organoids from CeD patients is reduced in the presence of probiotics from *Lactobacillus paracasei* and *Lactobacillus rhamnosus* GG (15–17).

Pro- and postbiotics and conjugated linoleic acids (CLAs) are helpful in treating inflammatory-related diseases (18–20). CLAs are polyunsaturated acids and are a mixture of positional and geometric isomers of linoleic acid, a polyunsaturated omega-6 fatty acid also known as cis, cis-9,12-octadecadienoic acid. Linoleic acid and CLA are essential fats that must be part of the diet. The concentration of CLAs can be increased naturally by the selection of *ad hoc* microorganisms and fermentation conditions. Linoleic acid is a precursor in the synthesis of CLA; it is often added to the matrix to be fermented in its pure form (21, 22) or by adding vegetable oils, such as safflower oil (23) and sunflower oil (24). Interestingly, the line guide written by Vitale and Giacco in 2020 defined roasted coffee waste, such as spent coffee grounds (SCGs), as foods which contains a high concentration of linoleic acid (25).

SCGs are the most abundant coffee byproduct (26), and in 2017, its worldwide production reached 9.6 million tons as described in International Coffee Organization (ICO). SCGs have been widely studied as a material for biodiesel production (27). However, in recent years, it has also been used for food applications (26).

The aims of the present work was both the sustainable production of a functional milk-based beverage fermented by *L. plantarum* CECT 749 that was enriched in CLAs after the addition of LA, SCGs and SCG oil, and the study of the potential anti-inflammatory effects of these compounds in the presence of p31-43 in Caco-2 cells and CeD intestinal organoids.

## 2 Materials and methods

### 2.1 Fermentation experimental apparatus

The system consisted of a Pyrex batch reactor (20 cm, ID of 10 cm, 1.5 L) equipped with an external jacket to circulate a service fluid from a thermostatically controlled water bath. The mixing system consisted of a stainless-steel impeller equipped with three Rushton turbines. It was connected to a motor that adjusted the stirring speed. The head plate was equipped with an input for the insertion of an In Pro 3,100 probe (Mettler Toledo, Milan, Italy) that was connected to an M300 transmitter (Mettler Toledo, Milan, Italy), which is helpful for inline temperature/pH measurements, and an input connected to a tank containing a 4 M NaOH solution that was fed by a peristaltic pump for pH control.

### 2.2 Strain

*Lactobacillus plantarum CECT 749* (from the Spanish Type Culture Collection, CECT, Valencia Spain) is a gram-positive bacterium that has been studied for its antifungal activity (28) and ability to produce CLA (21, 23, 29). The strain was stored at −20°C in cryovials containing 1 mL of a solution of animal-free broth [AFB- Animal Free Broth; composition: 20 g/L Bacto Yeast Extract (BD Biosciences, Milan, Italy), 0.5 g/L MgSO_4_ (Sigma–Aldrich, Milan, Italy), 50 g/L glucose (Sigma–Aldrich), 0.5 g/L citric acid (Sigma–Aldrich) and glycerol (20%, Sigma–Aldrich)]. Before each fermentation, after thawing, the strain was revitalized by adding 9 mL of AFB and then incubated for 24 h at 37°C. The revitalized strain had a bacterial concentration of 10^8^ CFU/mL, and the inoculum contained 1% v/v fermentation substrate.

### 2.3 Fermentation substrates and experimental conditions

The fermentation substrate was 1 L of skim milk (GranaroloⓇ - UHT), which was purchased from a local market and contained 20 g/L glucose (Sigma–Aldrich, Milan, Italy). To test the microbial capacity in the production of CLA, different compounds, as CLA precursors, were used, allowing the following experimental conditions (30) to be defined:

- Exp. Condition 1 (milk): Milk with only glucose as a negative control.
- Exp. Condition 2 (milk + LA): Milk with glucose and 0.5 mg/mL free linoleic acid (LA, 99% purity; Sigma Aldrich, St. Louis, MO, USA), since a previous study suggested this concentration as the best concentration for maximum CLA production (31).
- Exp. Condition 3 (milk + SCG oil): Milk with glucose and 1.3 g of the oil extracted from *Passalacqua^Ⓡ^* spent coffee grounds (*Passalacqua^Ⓡ^*, *Mexico* blend type, 100% Arabica) to guarantee that the free LA (from now on LA) concentration was the same as that in Exp. Condition 2 (where the LA concentration in spent coffee grounds corresponds to 40% of the total fat in the spent coffee ground oil) (32). The extraction method used to obtain the coffee oil is described below. “Free LA” are the linoleic acids totally available for the conversion in a conjugated form.
- Exp. Condition 4 (milk + SCG): Milk with glucose and 20 g of *Passalacqua^Ⓡ^* spent coffee grounds; this amount was chosen since the literature reported that, to obtain the optimal LA concentration (0.5 mg/mL), 10 grams of dry waste would be needed. Wet spent coffee grounds contain an average of 50–60% water; therefore, to release the abovementioned amount of LA, two times the weight (20 grams) of spent coffee grounds was added (32).

Glucose was added for each experimental condition to favor a more rapid fermentation process. The addition of linoleic acid, SCG oil, and raw SCGs to milk was followed by sonication to homogenize the matrix to be fermented and promote the dispersion and extraction of the fat phase in the dairy matrix.

For each condition tested, fermentation was carried out for 48 h at 37°C. Fermented samples were withdrawn aseptically from the reactor at specific times (after inoculation (t_0_) and after 24 (t_24_) and 48 (t_48_) hours).

### 2.4 SCG oil extraction method

SCG oil extraction was performed using a hydrofluorocarbon (HFC) solvent, 1,1,1,2-tetrafluoromethane (Norflurane, R134a), under subcritical conditions with a laboratory-scale system via a patented process (51). Norflurane is a haloalkane refrigerant with a boiling point of 26.3°C at atmospheric pressure and a vapor pressure of 6.61 bar at 25°C (33). Liquid Norflurane was percolated through a dry SCG matrix bed and placed in an extraction chamber at 8–10 bar to enrich its coffee oil content. The mixture was subsequently passed through an expansion vessel, where Norflurane was gasified at lower pressure (approximately 4–5 bar), and the oily solute was released at the bottom. Afterwards, the clean gaseous Norflurane was recompressed and recycled in liquid form to the extraction chamber, where the extraction phase was repeated.

### 2.5 Analytical methods

#### 2.5.1 Microbiological analysis

A spread plate method was used to determine the vital bacterial load of the inoculum, the microbial concentration during the entire process, and the presence of contaminants. After serial dilutions, the sample was spread on MRS agar (Oxoid, Basingstoke, UK) for the enumeration of lactobacilli; MacConkey agar (Oxoid, Basingstoke, UK) and Gelatin peptone agar (Biolife, Milan, Italy) were used for the detection of eventual contaminants. All the plates were incubated at 37°C for 48 h before being read. Anaerobic kits (Anaerogen Compact, Oxoid, Basingstoke, UK) were used for MRS plates to guarantee anaerobic growth conditions for *L. plantarum CECT 749* during incubation.

#### 2.5.2 Lactic acid quantification

The concentration of lactic acid was determined by high-performance liquid chromatography (HPLC) on an Agilent Technologies 1,100 instrument equipped with an Agilent Synergi Hydro-RP C18 column (250 mm × 4.6 mm and a pore size of 4 μm) and a visible/UV detector. The mobile phase consisted of 0.27% KH_2_PO_4_ aqueous solution at a pH of 2 modified with H_3_PO_4_ (eluent A) and 100% methanol (eluent B), and a gradient consisting of 30% B in 2.6 min followed by 100% A in 2.9 min with a flow rate of 1 mL/min was used. The detection wavelength was set at 210 nm.

#### 2.5.3 CLA analysis

Fat extraction was performed according to Stefanov et al. (33). A total of 10 g of milk fermented samples (collected at times t0, t24, t48 and postbiotics for each tested condition) was mixed with 16 mL of previously prepared dichloromethane–ethanol (DM–E) solution (2:1 ratio, v/v) in a 40 mL centrifuge-grade glass test tube, vortexed for 90 s and then centrifuged for 8 min (2,500 g at −4°C, MPW Med. Instruments, MPW-352R, Warszawa, Polonia). The supernatant was discharged, and 10 mL of the DM-E solution was added to the test tube. The mixture was vortexed and then centrifuged for 6 min (2,500 × g at −4°C). The upper organic phase containing the milk fat was filtered (597½, 240 mm diameter, Schleicher & Schuell, Dassel, Germany), dichloromethane was removed by a reduced pressure rotary evaporator (Bibby Sterilin RE-100, Bibby Scharlau Italia S.r.l., Riozzo di Cerro al Lambro (MI) Italia), and the samples were evaporated until dryness was achieved. After extraction, the fats were transesterified according to the method of Ichihara et al. (52). The lipid sample was dissolved in 0.20 mL of toluene, and then, 1.50 mL of methanol and 0.30 mL of the reagent mixture (consisting of 9.7 mL of HCI (35%, w/w) diluted with 41.5 mL of methanol) were added in this order. The tube was vortexed and then heated at 100°C for 1 h. After cooling, 1 mL of hexane and 1 mL of water were added to extract the methyl esters in the hexane phase. Finally, the samples were analyzed via GC (Varian CP 3800 Gascromatographer, Palo Alto, United States) equipped with an FID detector and a ZB WAX plus column (60 m 0.25 mm 0.2 μm, Phenomenex, Aschaffenburg, Germany), with helium used as the gas carrier (1.2 mL/min).

### 2.6 Caco-2 cells and treatment

Human colon adenocarcinoma-derived cells (Caco-2) obtained from the Interlab Cell Line Collection (Centro di Biotecnologie Avanzate, Genoa, Italy) were grown in DMEM supplemented with 10% fetal calf serum, 100 units of penicillin–streptomycin/mL, and 1 mmol/L glutamine (Gibco Invitrogen, Milan, Italy). The cells were maintained in a humidified atmosphere (95% air and 5% CO2) at 37°C. The postbiotic milk-based beverages were obtained by thermal treatment (85°C, 30 s). The milk-based postbiotic beverages were freeze-dried, resuspended in DMEM-free medium (2% w/v), centrifuged thrice at 1500 rpm for 5 min and after each centrifugation the supernatant was recovered, and after the last centrifugation, the supernatant was filtered using the 0.22 μm filter (ref SLGS033SS, Merk, Ireland). The sequence of P31-43, an LPS-free synthetic peptide, was LGQQQPFPPQQPY (Caslo > 95% purity, MALDITOF analysis; DK-2800 Kongens Lyngby, Denmark), and it was used at 100 μg/mL (15, 16). Previous studies describe the optimal concentration of p31-43 on Caco-2 cells to be 100 μg/mL, in these studies this concentration was able to have several different effects (6, 34).

For the treatment, more than 2×10^6^ cells were seeded in 60 mm culture dishes overnight for attachment. The next day, the cells were exposed in presence or not to milk-based probiotics and postbiotics supplemented with LA, SCG and SCG for 1 h, as based on previous published data. Caco-2 cells were pretreated with milk-based postbiotics at 2% w/v for 30 min and subsequently stimulated with P31–43 at 100 μg/mL (15, 16, 35).

#### 2.6.1 Cell viability and optimal concentrations of milk-based postbiotic

For the cell viability experiment, more than 2×10^6^ cells were seeded in 60 mm culture dishes overnight for attachment. The next day, the cells were exposed in presence or not to milk-based probiotics and postbiotics supplemented with LA for 24 and 48 h of fermentation at concentrations between 0.1 and 10% for 1 h at 37°C in DMEM-free medium. At the end of incubation, cell viability was assessed by a trypan blue dye exclusion assay based on the microscopic quantitation of live cells (unstained) and dead cells (blue cytoplasm). Cells were automatically counted using the Countess automated cell counter (Thermo Fisher Scientific, Milan, Italy). A high viability range was evident at concentrations of 0, 1, 1, and 2% (Supplementary Figures 1, 2). As the CLA concentration in the matrix studied was the same after 24 and 48 h of fermentation and considering that the viability did not change at 0.1% (1.3×10^7^ CFU/g), 1, and 2%, we used the postbiotics after 24 h at concentrations of 1% (1.3×10^8^ CFU/g) and 2% (2.6×10^8^ CFU/g) weight/volume.

### 2.7 Intestinal organoids

One to two duodenal biopsies were taken with standard endoscopic EGD (esophageal gastroduodenoscopy) from three control subjects, for which the final diagnosis is attributed to functional disorders, and three patients with CeD. The CeD patients was diagnosed following the ESPGHAN 2020 guidelines they presented villus atrophy in the intestinal biopsy (MARSH T3c) and were positive for anti-tTg antibodies in the serum. These conditions satisfied the ESPGHAN 2020 guidelines that state HLA-testing is not required in patients with positive TGA-IgA, if they qualify for CD diagnosis with biopsies or if they have high serum TGA-IgA ≥ 10xULN and EMA-IgA positivity. The biopsy samples were placed in 10 mL of ice-cold PBS supplemented with 2 mM EDTA (cat. 15,575,020, Thermo Fisher, Milan, Italy) and 0.5 mM DDT (D0632, Sigma–Aldrich, Milan, Italy). After 60 min, Crypt units were isolated by using washing buffer (WB) containing penicillin (100 units/mL), streptomycin (0.1 mg/mL), l-glutamine (2 mM), and FBS (10%, v/v) in DMEM/F12 with HEPES on ice for 30 min. The digest was filtered through a 70 m strainer (Falcon, Milan, Italy). Crypts were collected by centrifugation at 2800 rpm for 5 min. The supernatant was discarded, and the crypts were carefully resuspended in 40 μL of ice-cold Matrigel matrix (Corning 356,231, Milan, Italy) to enable three-dimensional growth in 48-well plates; the plates were incubated in a cell culture incubator at 37°C and 5% carbon dioxide for 10 min to allow the Matrigel to solidify. Afterwards, 300 μL of cell culture medium enriched with supplements (CM-S) (13) was added to each well and replaced every second day. For 2D organoids, organoids were seeded in six wells pretreated with Matrigel diluted at 1:40 in phosphate-buffered saline (PBS) (13, 15, 16, 36) (Table 1).

**Table 1:** Organoids characteristics.

Patients	Age range (Years)	Sex	Biopsy (marsh classification*)	Serum antiTG2 (U/mL)	Anti-endomysial antibody (EMA)
CTR (*n* = 3)	4–15	M = 2 F = 1	3 = T0	Negative	Negative
GCD-CeD (*n* = 3)	3–17	M = 2 F = 1	3 = T3c	>100	Positive

*T3: Flat destructive lesion (c: total).

The sex classification: M represent the male group, F represent the female group.

The serum antibody antiTG2 is Anti-Transglutaminase-2.

### 2.8 Western blot

After treatment, the Caco-2 cells were washed twice with cold PBS and resuspended in lysis buffer (50 mM Tris–HCl (pH 7.4), 1 mM EDTA, 1 mM EGTA, 5 mM MgCl_2_, 150 mMNaCl, 1% Triton, 1 mM PMSF, 1 mM VO4, 100 × Aprotinin, and 50 × LAP, all of which were purchased from Sigma, Milan, Italy, except for LAP, which was obtained from Roche, Milan, Italy). The cell lysates were analyzed using SDS-PAGE with a standard running buffer (25 mM Tris, 192 mM glycine, and 0.1% SDS) and transferred onto nitrocellulose membranes by a Trans-Blot Turbo transfer system (cat. 1704158; Bio-Rad, Milan, Italy) in a stain free gel at 10% (#cat 4,568,033; Bio-Rad, Milan, Italy). The membranes were washed, blocked with 5% non-fat dry milk, and probed with onto nitrocellulose membranes (Whatman Gmbh, Dassel, Germany). The membranes were blotted with rabbit anti-pNF-kB (Elabscience, Microtech, Naples, Italy) and mouse anti-GAPDH (Glyceraldehyde-3-phosphate dehydrogenase) (cat. G8795, Sigma-Aldrich). GAPDH or total protein intensity were used as loading controls. The bands were visualized using enhanced chemiluminescence (ECL) plus (Cat. 1705062 Bio-Rad Milan, Italy) with 2–10 min exposure times. The band intensity was evaluated by integrating all the pixels of a band after subtraction of the background to calculate the average of the pixels surrounding the band (13, 15, 16, 36).

### 2.9 ELISA

The levels of IL-8 were measured using commercial test kits (IL-8/CXCL8 cat.#D8000C, Bio-techne R&D System, Milan, Italy) on culture media of GCD-CeD intestinal organoids treated and not with milk-based *L. plantarum* of postbiotics with and without SCG respect to CTRs’.

### 2.10 Statistical analysis

Statistical analysis and graphics were obtained from Graph Pad Prism (San Diego, CA, USA). The means and standard deviations of the experimental data were calculated; their significance was evaluated by Student’s *t* test and two way ANOVA multiple comparison, with only results that presented values of *p* < 0.05 considered significant.

## 3 Results

### 3.1 Spent coffee grounds (SCGs) improved bacterial growth and lactic acid production

**Figure 1:**
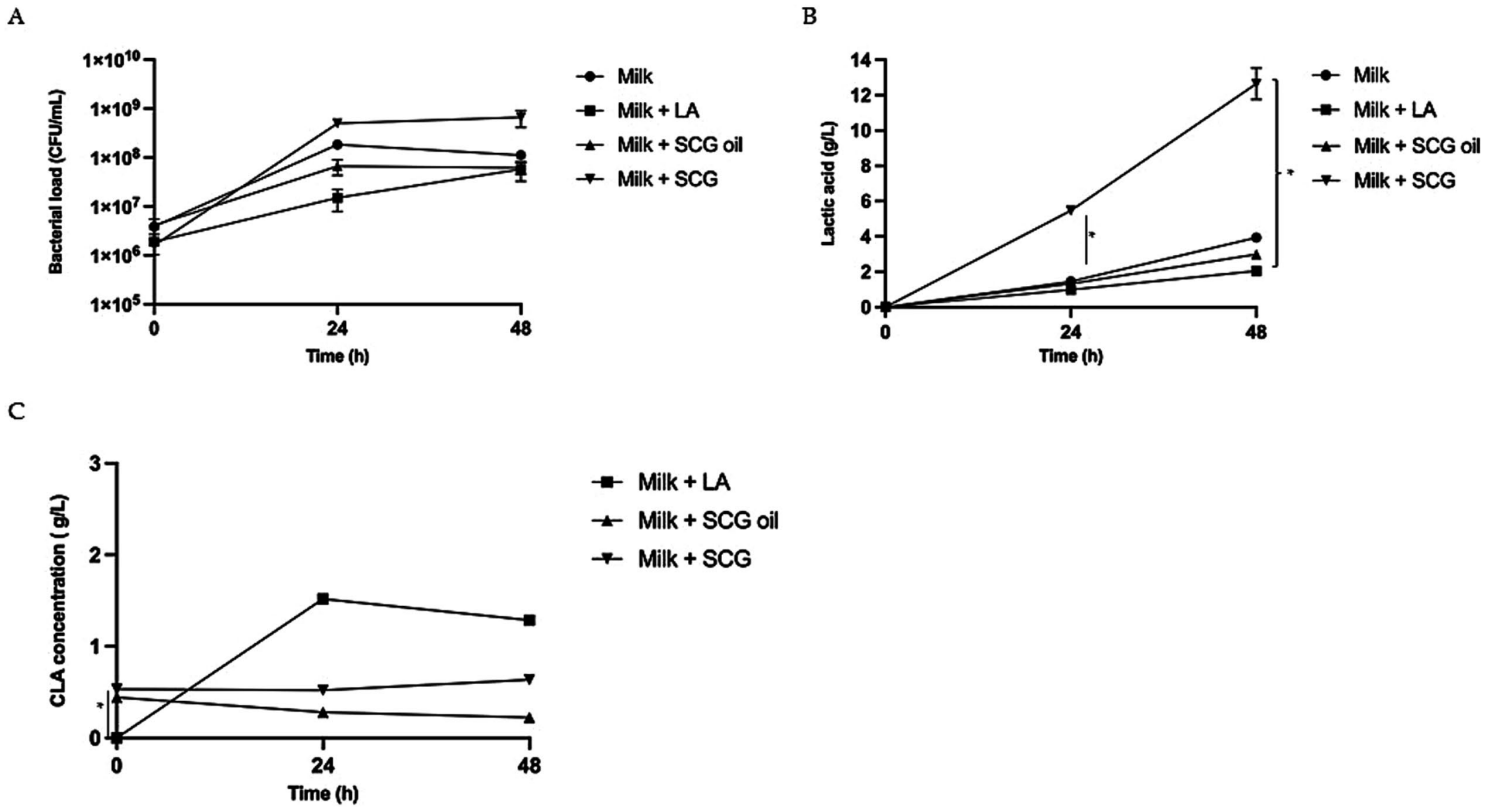
Bacterial growth, lactic acid production and CLAs concentration in milk-based postbiotics with linoleic acid (LA), Spent Coffee Grounds (SCGs) and SCG oil. **(A)** Bacterial growth (CFU/mL) observed after 0, 24 and 48 h of fermentation in milk alone (milk, circles), milk fermented with linoleic acid (milk + LA; squares), milk fermented with spent coffee ground oil (milk + SCG oil; triangles) and milk fermented with raw spent coffee grounds (milk + SCGs; inverted triangles). The bars represent the standard deviations of three independent experiments. Different letters indicate significant differences. ANOVA test: * = *p* < 0.05. **(B)** Lactic acid production observed after 0, 24 and 48 h of fermentation in milk alone (milk, circles), milk fermented with linoleic acid (milk + LA; squares), milk fermented with spent coffee ground oil (milk + SCG oil; triangles) and milk fermented with raw spent coffee grounds (milk + SCGs; inverted triangles). The bars represent the standard deviations of three independent experiments. Different letters indicate significant differences ANOVA test: * = *p* < 0.05. **(C)** Concentration of total CLAs (g/L) observed after 0, 24 and 48 h of fermentation in milk fermented with linoleic acid (Milk + LA; squares), milk fermented with spent coffee ground oil (milk + SCG oil; triangles) and milk fermented with raw spent coffee grounds (milk + SCGs; inverted triangles). The bars represent the standard deviations of three independent experiments. Different letters indicate significant differences ANOVA test: * = *p* < 0.05.

We studied Plantarum CECT 749 bacterial growth under different conditions (Figure 1). In all cases, the initial concentration was approximately 10^6^ CFU/mL, reaching 1.8×10^8^ ± 2.6×10^7^ CFU/mL after 24 h of fermentation in the case of the negative control (milk); 1.5×10^7^ ± 7.1×10^6^ CFU/mL when milk was supplemented with linoleic acid (milk +LA); 6.7×10^7^ ± 2.3×10^7^ CFU/mL when milk was supplemented with spent coffee ground oil (milk + SCGs oil); and 5.0×10^8^ ± 7.1×10^7^ CFU/mL when milk was supplemented with raw spent coffee grounds (milk + SCGs) (Figure 1A).

Milk supplemented with SCGs resulted in the best matrix for *L. plantarum* growth, reaching 6.6×10^8^ ± CFU/mL at 48 h of fermentation, a value that was one log higher than that observed for milk fermented with LA (5.8×10^7^ ± 2.5×10^7^ CFU/mL) and that observed for milk fermented with SCG oil (6.2×10^7^ ± 1.6×10^7^ CFU/mL) (Figure 1A).

The amount of lactic acid produced in fermented milk (Figure 1B) in the presence of SCGs was 5.46 ± 0.05 g/L after 24 h (*p* < 0.05 respect to Milk, Milk with LA and Milk with SCG oil) and 12.66 ± 0.88 g/L after 48 h (p < 0.05 respect to Milk with LA) of fermentation. Milk fermented with only glucose (negative control) showed lactic acid production (lactic acid at 24 h: 1.46 ± 0.12 g/L; at 48 h: 3.94 ± 0.10 g/L) amounts that were similar to that measured in milk fermented with LA (lactic acid at 24 h: 0.98 ± 0.22 g/L; at 48 h: 2.05 ± 0.24 g/L) and with SCG oil (lactic acid at 24 h: 1.32 ± 0.13 g/L; at 48 h: 2.99 ± 0.15 g/L).

### 3.2 LA supplementation of milk promoted CLA production during fermentation with *Lactobacillus plantarum*

**Figure 2:**
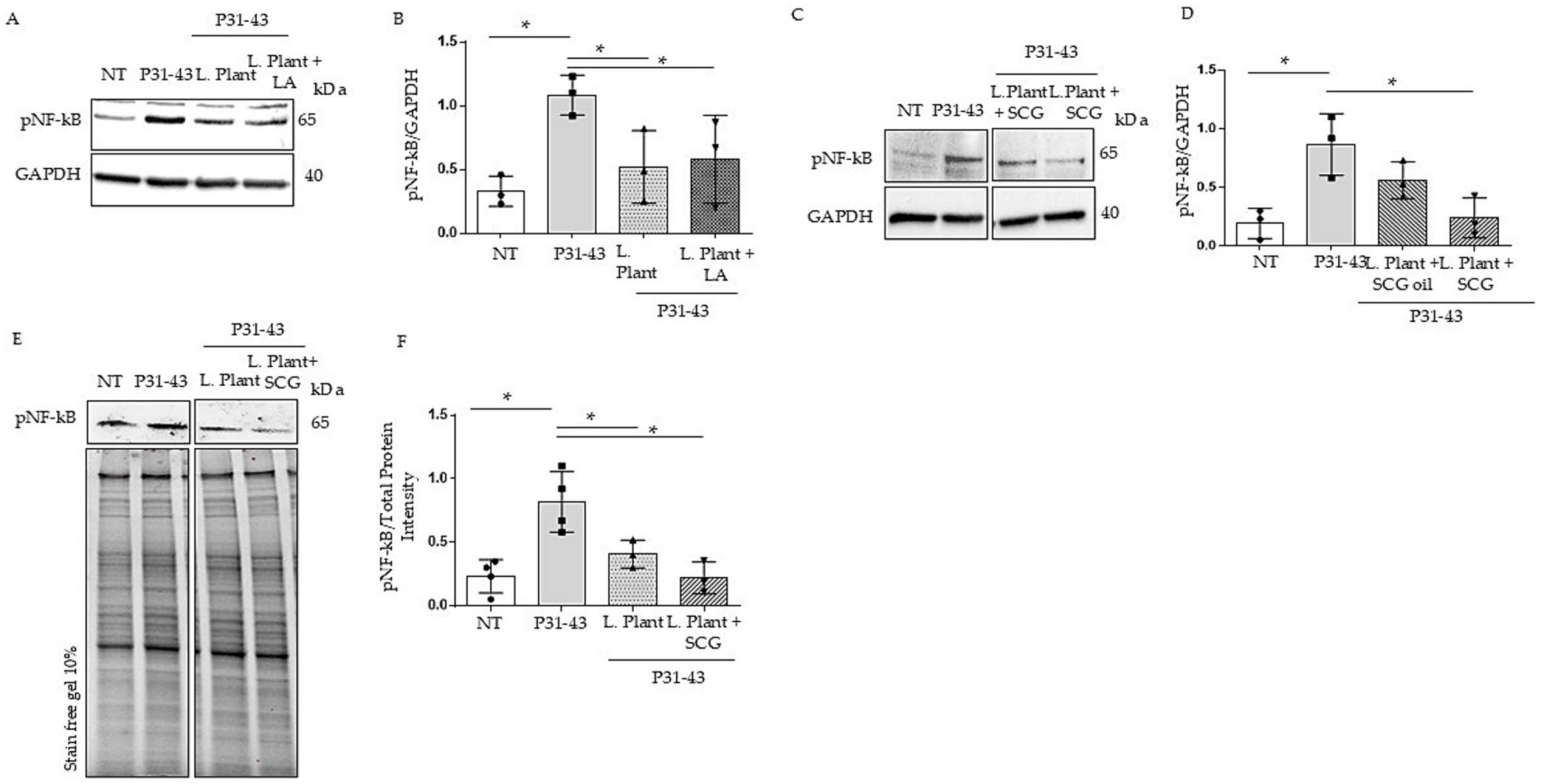
Effects of milk based postbiotics of *L. plantarum* CECT 749, with or without the addition of LA, SCG oil or SCGs at 2% concentration, after 24 h of fermentation on Caco-2 cells after treatment with P31-43. **(A)** Western blot analysis of protein lysates from untreated Caco-2 cells (NT), those treated with P31-43 for 1 h, and those pretreated with milk based postbiotic of *L. plantarum* CECT 749 after 24 h of fermentation with the addition of LA, blotted with antibodies against pNF-kB. GAPDH was used as a loading control. The immunoblotting analysis was representative of three independent experiments. **(B)** Densitometric analysis of bands from the WB shown in **A**. Columns represent the mean, and bars represent the standard deviation of the relative intensity of pNF-kB with respect to the total GAPDH protein. Student’s *t* test: * = *p* < 0.05. **(C)** Western blot analysis of protein lysates from untreated Caco-2 cells (NT), those treated with P31-43 for 1 h, and those pretreated with milk-based postbiotic of *L. plantarum* CECT 749 after 24 h of fermentation with the addition of SCG oil and SCGs, were blotted with antibodies against pNF-kB. GAPDH was used as a loading control. The immunoblotting analysis was representative of three independent experiments. **(D)** Densitometric analysis of bands from the WB shown in **A**. Columns represent the mean, and bars represent the standard deviation of the relative intensity of pNF-kB with respect to the total GAPDH protein. Student’s *t* test: * = *p* < 0.05. **(E)** Western blot analysis of protein lysates from untreated Caco-2 cells (NT), those treated with P31-43 for 1 h, and those pretreated with milk based postbiotic of *L. plantarum* CECT 749 after 24 h of fermentation, and alone and with the addition of SCGs, were blotted with antibodies against pNF-kB. Stain-free gel was used as a loading control. The immunoblotting analysis was representative of three independent experiments. **(F)** Densitometric analysis of the bands from the WB shown in **A**. Columns represent the mean, and bars represent the standard deviation of the relative intensity of pNF-kB with respect to the total protein intensity. Student’s *t* test: * = *p* < 0.05.

The concentration of total CLAs found during fermentation is shown in Figure 2. SCG oil and SCGs, which were used as sources of linoleic acid, were tested for CLA production. CLAs were present at T0 in the samples containing SCG and SGC oil even before fermentation, with concentrations of approximately 0.5 g/L for both samples. The difference was statistically significant for both SCG and SGC oil respect to the sample milk added with LA (*p* < 0.05 for both matrix) (Figure 1C). Milk fermented alone is not represented in the graph because no CLAs were detected, as expected. Milk fermentation in the presence of LA was the only sample in which CLAs were produced, with concentrations of 1.52 ± 0.06 g/L (*p* < 0.05) and 1.29 ± 0.04 g/L (*p* < 0.05) after 24 h and 48 h, respectively. The CLA concentration in the different tested postbiotics remained approximately the same as that evaluated in the probiotic samples confirming that the mild heat treatment did not affect the CLA concentration.

### 3.3 Pretreatment of milk-based postbiotics with or without the addition of LA or SCGs prevented the P31-43-induced increase in pNF-kB

We tested the effects of milk-based postbiotics on Caco-2 at concentrations of 1 and 2%, because these were able to preserve the cell viability (Supplementary Figures 1, 2). Both 1% (Supplementary Figure 1) and 2% concentrations were used to prevent on NF-kB increase mediated by p31-43, but only the 2% w/v of the postbiotics (2.6×10^8^ CFU/g) was able to prevent p31-43 effects on NF-kB activation (Figures 2A,B). Indicating that the concentration of the post biotic is an important factor for its activity. Caco-2 cells were treated with milk-based postbiotics with the addition of LAs to understand whether CLAs exert different biological effects on inflammation pathways. Caco-2 cells were pretreated with milk-based *L. plantarum* postbiotics at 2% w/v, with or without the addition of LA, for 30 min, and subsequently stimulated with p31-43. To assess inflammation, we evaluated the phosphorylation of NF-kB by WB. Our data revealed an increase in pNF-kβ levels after stimulation with P31-43. Both milk-based postbiotics (with and without the addition of an LA source) reduced the level of pNF-kβ induced by p31-43. These findings indicated that milk-based postbiotics with LA achieved the same pNF-kβ reduction effect as milk-based postbiotic with only *L. plantarum* (Figures 2A,B, *p* < 0.05 for both).

Additionally, we evaluated the effects of milk-based postbiotics added with SCGs and SCG oil in Caco-2 cells in the presence of the gliadin peptide. For this purpose, Caco-2 cells were pretreated with milk-based postbiotics at 2% w/v for 30 min and subsequently stimulated with P31-43, and the activation of pNF-kβ was evaluated via WB. Pretreatment with milk-based postbiotics combined with SCGs reduced in a statistically significant way the levels of pNF-kβ induced by p31-43 (Figures 2C,D, *p* < 0.05). However, milk-based postbiotics with SCG oil did not significantly inhibit the increase in pNF-kβ levels induced by P31-43 (Figures 2C,D).

The milk-based postbiotic with SCGs was more efficient than the milk-based postbiotic in preventing P31-43-induced activation of NF-kB under the same conditions (Figures 2E,F). In fact, the mean phosphorylation of NF-kB in Caco-2 cells pretreated with the milk-based *L. plantarum* postbiotic with SCGs was half than that of NF-kB in Caco-2 cells pretreated with the milk-based *L. plantarum* postbiotic, although a significant difference was not detected between the two groups analyzed.

### 3.4 In CeD intestinal organoids milk based postbiotic of *Lactobacillus plantarum* with or without SCGs reduced inflammation

The milk-based postbiotic activity of *L. plantarum* was tested in intestinal organoids derived from CeD patients with villus atrophy and compared with that of intestinal organoids derived from control subjects. Intestinal organoids can be obtained from stem cells LGR5 + present in intestinal crypts. This kind of multipotent cells could differentiate toward a limited number of cell types in the presence of specific growth factors. The stem cells are grown in 3D and enclosed in a matrix, which serves as a basal membrane. By using appropriate stimuli, they differentiate into epithelial cells. They can be grown and amplified for several weeks as spheroids. However, to know the response to stimuli of a pro-inflammatory nature or to investigate the possible beneficial effect of pre-pro-postbiotics and nutraceuticals or drugs, it is necessary to create a 2D monolayer of intestinal epithelial cells. Two-dimensional monolayers of intestinal organoids allow direct contact of the apical, adsorptive pole of the cells with the culture medium; this cannot occur in 3D culture as the apical sides of the cells are enclosed in the spherical organoids. Due to the lack of an appropriate scaffold, they do not form villi, instead the crypts are nicely represented as budding from the main spheroid (Supplementary Figure 2). Morphologically they do not show great differences although we (13) and others (37) have noticed that GCD-CeD organoids appear more dense respect to controls.

**Figure 3:**
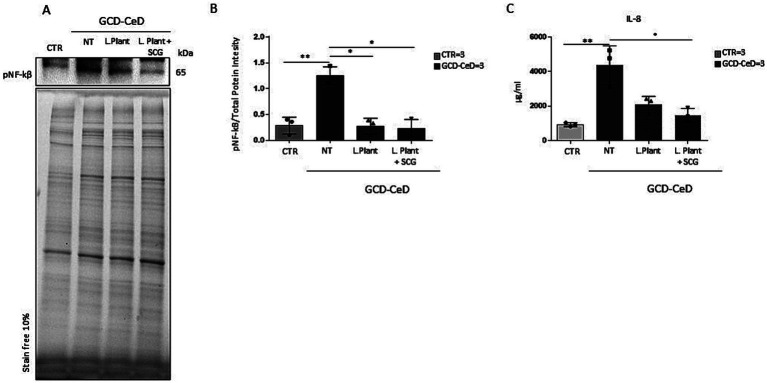
Effects of the milk-based postbiotic of *L. plantarum CECT 749* and milk-based postbiotic of *L. plantarum* alone or with the addition of SCGs in intestinal organoids of CeD patients. **(A)** Western blot analysis of protein lysates from organoids from CeD patients, untreated and treated with milk-based postbiotic of *L. plantarum* alone or with the addition of SCGs for 3 h, and control organoids were blotted with antibodies against pNF-kB. **(B)** Densitometric analysis of the bands from the WB shown in **A**. Columns represent the mean, and bars represent the standard deviation of the relative intensity of pNF-kB with respect to the total protein intensity. Number of organoids was indicated. Student’s *t* test: * = *p* < 0.05. **(C)** ELISA showing IL-8 cytokine levels in the culture media of GCD-CeD intestinal organoids treated with and without milk-based postbiotics of *L. plantarum*, with and without SCG and CTR organoids. The numbers of organoids analyzed are indicated. The columns represent the media, and bars represent the standard deviation. Student’s *t* test: * = *p* < 0.05, ** = *p* < 0.05.

To test postbiotic activity, intestinal organoids were opened in 2D on Matrigel pretreated plates and treated with milk-based postbiotics from *L. plantarum* with or without SCGs for 3 h. Compared with CTRs organoids, intestinal organoids from CeD patients were inflamed (Figures 3A,B, *p* < 0.01). In fact, CeD organoids presented increased NF-kB phosphorylation levels, as measured by WB^13^. Compared with that of the untreated CeD intestinal organoid, the pNF-kB of the milk-based *L. plantarum* postbiotic with or without the addition of SCGs was reduced by 4.63- and 5.42-fold, respectively (Figures 3A,B, *p* < 0.05 for both). Interestingly, the levels of pNF-kB in intestinal organoids from CeD patients after treatment with milk-based *L. plantarum* postbiotics with and without SCGs were similar to the levels found in intestinal organoids from controls.

IL-8 an inflammatory cytokine most abundant in the intestinal epithelium can induce NF-kB activation (38) and is increased in IBD and CeD intestinal biopsies (39). Culture media from CTR and GCD-CeD, treated and not with milk- based *L. plantarum* postbiotics with and without the addition of SCG were analyzed by ELISA assay to test IL-8 levels. The results shown in Figure 3C indicate that IL-8 increased in CeD respect to CTR in a statistically significant way. The treatment with both the postbiotics, with and without, SCG was able to decrease IL-8 levels, but only the postbiotic with SCG reduced this cytokine in a statistically significant way, indicating that the SCG on some read outs is more efficient than the postbiotic alone.

### 3.5 Discussion

We demonstrated that the presence of LA and SCG oil in the fermented milk negatively affected both bacterial growth and lactic acid production during 48 h of fermentation. On the other side the addition of SCG promoted microbial proliferation and lactic acid formation which were even higher than those observed in fermented milk without any additives. Indicating that the SCG probably is improving the bacterial growth and lactic acid formation. *L. plantarum* could convert LA to conjugated linoleic acids (CLAs). As a source of LA we used not only LA, but also the used food waste SCGs and SCG oil that are a natural source of LA (32). The maximum amount of CLAs was produced after 24 h of fermentation in milk supplemented with LA, with a higher CLA concentration being achieved than other concentrations reports in the literature where the same fermentation conditions (31) or the same microorganisms (21) were used. During fermentation in milk supplemented with SCGs or SCG oil, no CLA production was observed, probably because CLAs were already abundant in the matrix supplemented with the coffee byproducts.

The higher bacterial growth and lactic acid production observed when SCGs was added to milk could be due to the presence of some oligosaccharides (40), such as mannooligosaccharides (MOS) and galactomannans, for which previous works have already shown the prebiotic effect on lactic acid bacteria (41). SCGs has already been used as a substrate to be fermented by lactic acid bacteria to produce lactic acid (42, 43), after preliminary chemical and enzymatic pretreatments. In the present work, *L. plantarum* was able to metabolize SCGs and to use it as prebiotic without any pretreatment, to the best of our knowledge this has been unreported before.

SCGs are a rich source of polysaccharides (more than 50% by dry weight), such as cellulose and hemicellulose; among the sugars, mannose, galactose, and glucose are the most abundant. Lignin is also present in significant amounts (close to 25%), and the total fiber content is 60% (44). Approximately 20% of SCGs consist of proteins. SCGs have antioxidant potential (44) because of the presence of phenolic compounds (Supplementary Table 3) such as chlorogenic acid, caffeine, and flavonoids; considering its high fiber content, high concentration of linoleic acid and anti-inflammatory potential, it can be considered for the development of functional foods.

In the present study, we investigated the effects of milk-based postbiotics obtained from *L. plantarum* CECT 749 with and without free LA, SCGs or SCG oil on inflammation in Caco-2 cells after treatment with the gliadin peptide P31-43 and in intestinal organoids from CeD patients. Intestinal epithelial cells derived from intestinal carcinoma, Caco-2 cells, or staminal intestinal cells such as intestinal organoids were used to test the ability of milk-based postbiotics to prevent gliadin peptide P31-43-induced inflammation.

Caco-2 cells are widely used as an *in vitro* model to study the intestinal barrier and intestinal absorption in patients with celiac disease and gluten sensitivity (8, 34). After exposure to gluten peptides, Caco-2 cells exhibit increased intestinal permeability, and several other pathways, such as inflammation are altered (8). Conte et al. demonstrated that in the presence of both P31-43 and PTG, Caco-2 cells were inflamed and exhibited increased pNF-kB levels (15). In the same model, postbiotics from both *L. paracasei* and *L. rhamnosus* GG prevented the proinflammatory effects induced by gliadin peptides on Caco-2 cells (15, 16).

The effect was observed only with 2% fermented products, indicating that their activity is concentration dependent and that *L. plantarum* with added LA was able to prevent the effects of P31-43 on NF-kB activation. Although *L. plantarum* with SCGs was more efficient than *L. plantarum* alone in preventing P31-43 activity, the difference was not statistically significant. *L. plantarum* with SCG oil alone was not able to prevent the effects of P31-43 on CaCo2 cells. This is probably due to the fact that SCG oil does not benefit the bacteria’s growth, activities and the production of active metabolites. The interference of the fatty matrix on bacterial activities has already been described in the literature (45, 46).

Considering that three different postbiotic samples characterized by a different CLA concentration (namely milk with LA: 1,5 g/L CLA; milk without any addition: no CLA detected; milk with SCG: 0,5 g/L CLA) all showed a similar anti-inflammatory capacity, it is very likely that the effects on P31–43-induced inflammation did not depend on CLA production. We do not know why *L. plantarum* lost its activity after treatment with SCG oil; very likely, the high-fat matrix was not ideal for the production of one or more metabolites needed for the anti-inflammatory effect.

Intestinal organoids, which mimic miniature of the intestine, are a cell model used to study pathogenic agents and mechanisms related to celiac disease, such as inflammation (37, 47–49). Organoids are good models for studying some pathogenetic mechanisms of CeD, such as proliferation and constitutive inflammation, because they reproduce these alterations found in CeD biopsy samples (13, 36). Moreover, other studies demonstrated that treatment with *L. paracasei* and *L. rhamnosus* GG was able to reduce constitutive inflammation in CeD intestinal organoids (15, 16). In this manuscript we have confirmed that GCD-CeD organoids were inflamed respect to the controls by using two different read outs, namely the activation of NF-kB and the levels of IL-8 in the culture media. Both were increased in GCD-CeD organoids and decreased by the milk-based postbiotic with and without the addition of SCG. To note is the result on IL-8 reduction that was statistically significant only after treatment with milk-based *L. plantarum* postbiotic added with SCG.

The mechanism by which milk-based postbiotics obtained by the fermentation of *L. plantarum*, with and without the addition of LA or SCGs, exert their biological effects after treatment with P31-43 in Caco-2 cells and CeD intestinal organoids still needs to be clarified. The possible ability of milk-based postbiotics of *L. plantarum* with SCGs to prevent P31-43-induced inflammation could be associated with the presence of molecules with antioxidant activity and anti-inflammatory effects in this waste product (50). Further studies will be necessary to investigate which metabolites exert anti-inflammatory effects. Interestingly, in presence of SCG the bacteria grow was better and produce more lactic acids, indicating a better fermentation activity, but the production of CLA that was basically absent. All this indicating that CLA are not responsible for the anti-inflammatory activity, probably mediated by other metabolites. However, this observation confirms the possibility of reusing food waste from coffee processing to produce functional foods with improved beneficial properties. This enhanced effect of SCGs cannot be described on the constitutive inflammation of CeD organoids. This is probably because different inflammatory pathways are engaged in intestinal organoids.

In conclusion, milk-based postbiotics of *L. plantarum*, with or without SCGs, can prevent P31-43-induced inflammation in Caco-2 cells and reduce constitutive inflammation in CeD organoids.

These preclinical studies provide a good basis for initiating clinical trials in celiac patients to prevent the proinflammatory effects of gliadin peptides. It would be interesting to test the ability of postbiotics to prevent disease in potential patients who have anti-transglutaminase antibodies but have not yet developed the intestinal lesions typical of celiac disease. However, more studies are needed to investigate the related safety parameters and biological activity of postbiotics *in vivo*. Despite patients’ best efforts, some subjects can experience continuous exposure due to cross-contamination or traces of gluten in food. These risks could, in some cases, compromise the health and quality of life of these patients. Therefore, it is generally useful to study compounds that can prevent the inflammatory effects of gliadin with the hope of reducing the burden of living with celiac disease and improving long-term health outcomes.

## Data Availability

The original contributions presented in the study are included in the article/Supplementary material, further inquiries can be directed to the corresponding author.
