# Supplementary figures and images for: Sustainable milk-based postbiotics beverages fermented by Lactobacillus plantarum: allies in celiac disease inflammation
Source: Front Nutr. 2025 May 13;12:1549120. doi: 10.3389/fnut.2025.1549120 (PMC12107829; doi:10.3389/fnut.2025.1549120)

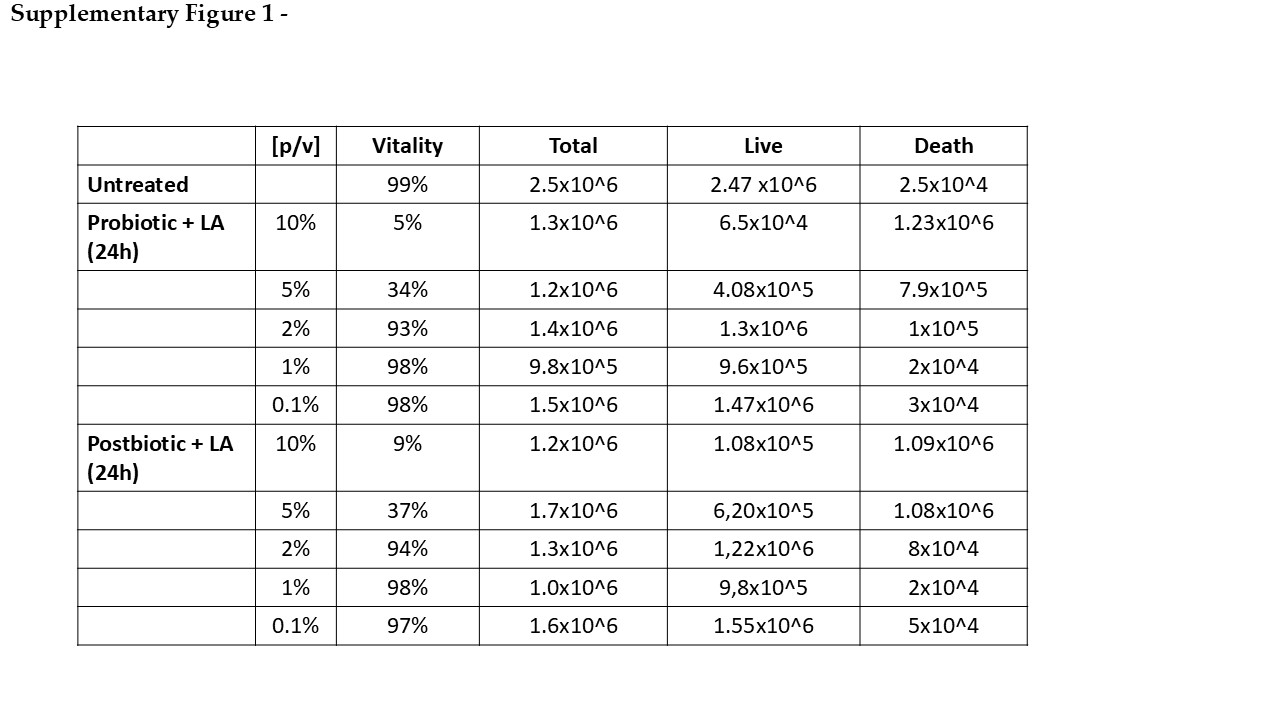

Supplement: Supplementary file 1 [file Image_1.jpeg]

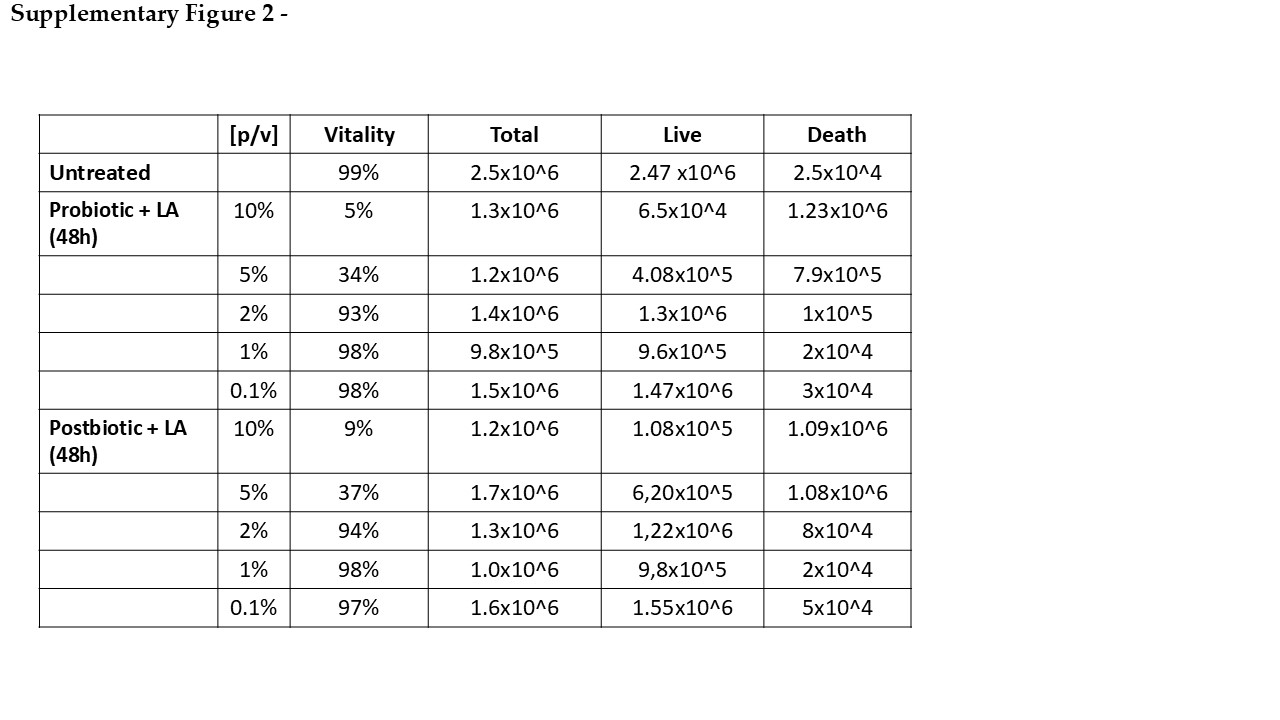

Supplement: Supplementary file 2 [file Image_2.jpeg]

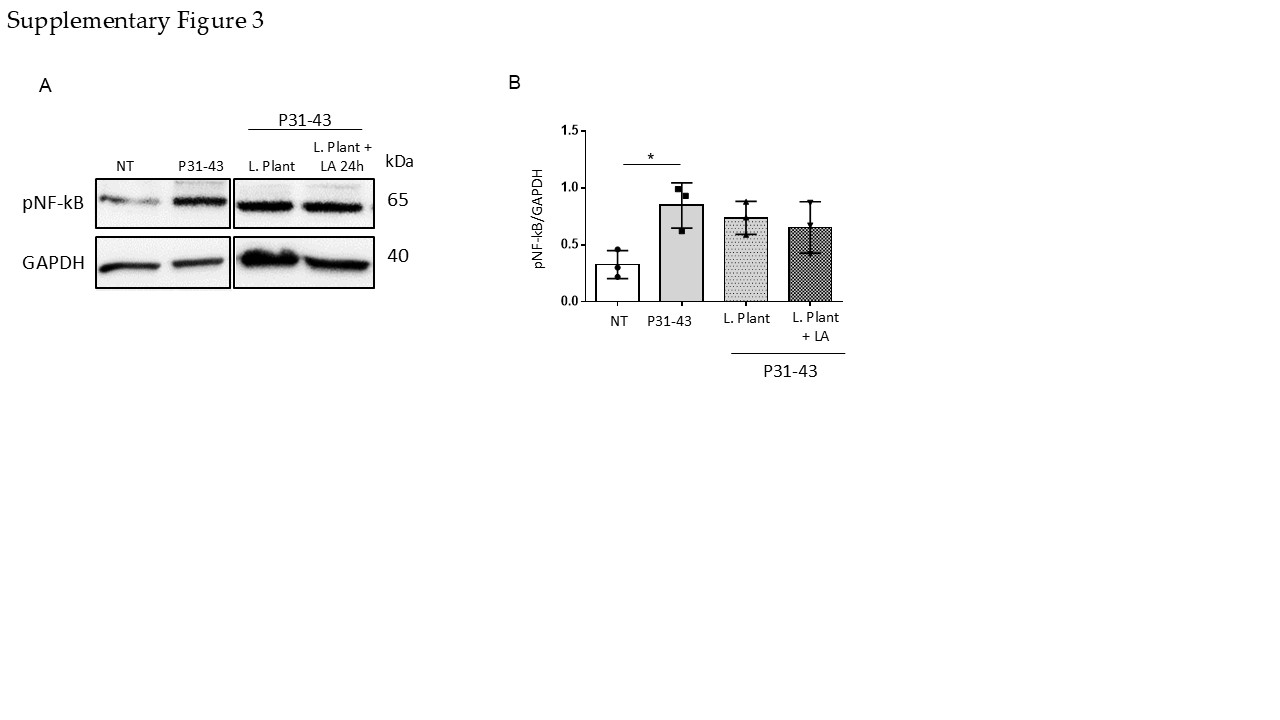

Supplement: Supplementary file 3 [file Image_3.jpeg]
