# Supplementary material for: Sustainable milk-based postbiotics beverages fermented by Lactobacillus plantarum: allies in celiac disease inflammation
Source: Front Nutr. 2025 May 13;12:1549120. doi: 10.3389/fnut.2025.1549120 (PMC12107829; doi:10.3389/fnut.2025.1549120)
Supplement: Supplementary file 4 [file Table_1.docx]

Supplementary Material

1. **Supplementary Table 1**

|  | **[p/v]** | **Vitality** | **Total** | **Live** | **Death** |
| --- | --- | --- | --- | --- | --- |
| **Untreated** |  | 99% | 2.5x10^6 | 2.47 x10^6 | 2.5x10^4 |
| **Probiotic + LA (24h)** | 10% | 5% | 1.3x10^6 | 6.5x10^4 | 1.23x10^6 |
|  | 5% | 34% | 1.2x10^6 | 4.08x10^5 | 7.9x10^5 |
|  | 2% | 93% | 1.4x10^6 | 1.3x10^6 | 1x10^5 |
|  | 1% | 98% | 9.8x10^5 | 9.6x10^5 | 2x10^4 |
|  | 0.1% | 98% | 1.5x10^6 | 1.47x10^6 | 3x10^4 |
| **Postbiotic + LA (24h)** | 10% | 9% | 1.2x10^6 | 1.08x10^5 | 1.09x10^6 |
|  | 5% | 37% | 1.7x10^6 | 6,20x10^5 | 1.08x10^6 |
|  | 2% | 94% | 1.3x10^6 | 1,22x10^6 | 8x10^4 |
|  | 1% | 98% | 1.0x10^6 | 9,8x10^5 | 2x10^4 |
|  | 0.1% | 97% | 1.6x10^6 | 1.55x10^6 | 5x10^4 |

## Supplementary table 1. Caco-2 cell survival evaluated after treatment, for one hour, with different concentration of milk based Probiotic and Postbiotics of L. Plantarum CECT 749 after 24 hours.

## Cell viability was assessed by trypan blue staining after one hour of exposure to the postbiotics in five different concentration from 10 to 0,1 [% p/v]. Untreated cells were used as negative controls.

|  | **[p/v]** | **Vitality** | **Total** | **Live** | **Death** |
| --- | --- | --- | --- | --- | --- |
| **Untreated** |  | 99% | 2.5x10^6 | 2.47 x10^6 | 2.5x10^4 |
| **Probiotic + LA (48h)** | 10% | 5% | 1.3x10^6 | 6.5x10^4 | 1.23x10^6 |
|  | 5% | 34% | 1.2x10^6 | 4.08x10^5 | 7.9x10^5 |
|  | 2% | 93% | 1.4x10^6 | 1.3x10^6 | 1x10^5 |
|  | 1% | 98% | 9.8x10^5 | 9.6x10^5 | 2x10^4 |
|  | 0.1% | 98% | 1.5x10^6 | 1.47x10^6 | 3x10^4 |
| **Postbiotic + LA (48h)** | 10% | 9% | 1.2x10^6 | 1.08x10^5 | 1.09x10^6 |
|  | 5% | 37% | 1.7x10^6 | 6,20x10^5 | 1.08x10^6 |
|  | 2% | 94% | 1.3x10^6 | 1,22x10^6 | 8x10^4 |
|  | 1% | 98% | 1.0x10^6 | 9,8x10^5 | 2x10^4 |
|  | 0.1% | 97% | 1.6x10^6 | 1.55x10^6 | 5x10^4 |

**Supplementary Table 2**

**Supplementary table 2. Caco-2 cell survival evaluated after treatment for one hour with different concentration of milk-based Probiotic and Postbiotics of L. Plantarum CECT 749 after 48 hours.**

Cell viability was assessed by trypan blue staining after one hour of exposure to the postbiotics in five different concentration from 10 to 0,1 [% p/v]. Untreated cells were used as negative controls.

**Supplementary Table 3**

|  | **SCG matrix**  (mgGAE/g dry solid) | **SCG oil**  (mgGAE/g oil) |
| --- | --- | --- |
| TPC | 6.28 ± 0.04^a^ | 5.95 ± 0.08^b^ |

**Supplementary table 3. Total polyphenol content (TPC) determined in the SCG solid matrix and SCG oil extracted by Norflurane.**

Values with different lowercase letters in the same row are significantly different (p<.05).

*GAE: gallic acid equivalents;*

*TPC was calculated using the Folin-Ciocalteu method (Colucci Cante et al., 2023)*

##
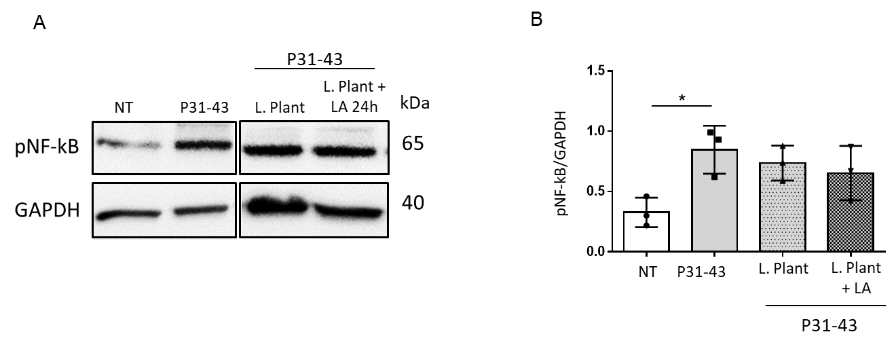
Supplementary Figures 1

**Supplementary figure 1. Effects of milk based postbiotic of *L. Plantarum CECT 749* with and without the addition of LA in Caco-2 cells in presence of P31-43 at 1% of concentration.**

(A)Western blot analysis of protein lysates from Caco-2 cells untreated (NT), treated with P31-43 for one hour, and pretreated with milk based postbiotic of L. Plantarum with and without the addition of LA were blotted with antibodies against pNF-kB. GAPDH was used as a loading control. The immunoblotting analysis was representative of three independent experiments. (B) Densitometric analysis of bands from WB as in A. Columns represent the mean, bars the standard deviation of the relative intensity of pNF-kB respect to total GAPDH protein. Student’s t-test = * p < 0.05, ** p < 0.01.

## Supplementary Figures 2


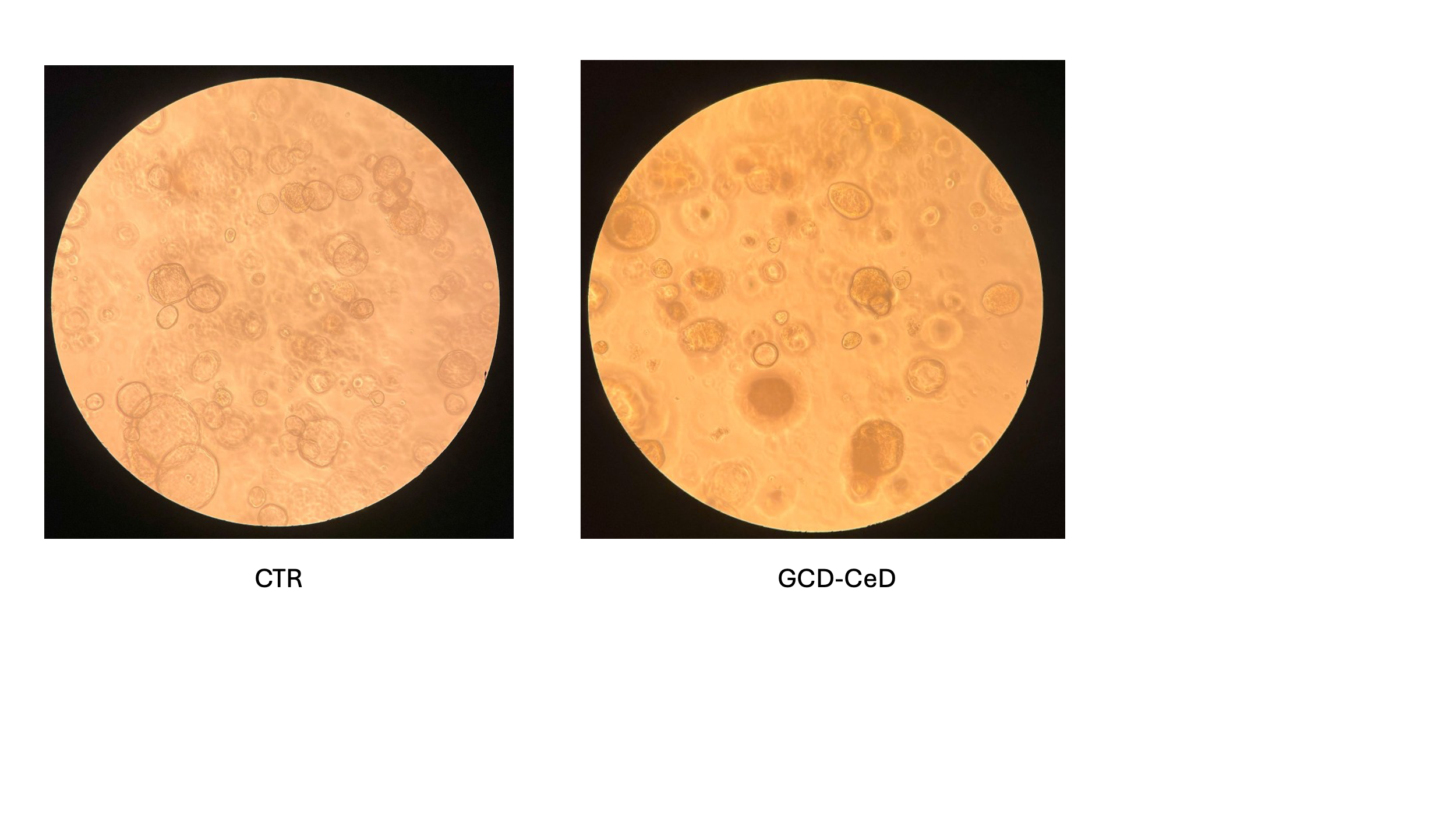


**Supplementary figure 2. Organoids from GCD-CeD patients are denser than CTR’s.** Images of 3D organoids colture from a control (CTR), on the left side and CeD patient on the right side. Black arrows indicate denser organoids in CeD sample. Direct light microscopy images (Zeiss Axiovert 40C), objective 10x.
